# P342: Patient safety and risk management in health center in Niger

**DOI:** 10.1186/2047-2994-2-S1-P342

**Published:** 2013-06-20

**Authors:** H Djibo, O Diakite, M Adamou

**Affiliations:** 1 Department of Public Health, Faculty of Health Sciences, Niamey, Niger; 2 Physician Health District, Niamey, Niger

## Introduction

The situation of adverse events related to health care in Niger like developing countries (DCs) remains poorly studied and therefore poorly managed.

## Objectives

For the hospital, it is a challenge for the future to the extent that this approach aims to: improve the safety of patients and staff, preserve the image of the institution, reduce the direct impact of risks and associated costs, protect workers against the risk of litigation.

## Methods

We performed at the National Hospital Lamordé, a prospective cross-sectional study. Pediatric services, pneumophtisiology, urology, pediatric surgery, emergency and we have served as framework for this study. In this survey we asked health workers and users of the hospital. Then we observed working sessions of participants.

## Results

This work has revealed that 60% of workers have experienced patients who experienced adverse events during their hospital stay, 65.7% have once had an accident with exposure to blood. Hand washing is not systematic in 45% of cases, the disposable equipment was sometimes used in 25.7%. It was also found that immunization against hepatitis B was very poor 14.3%, while blood exposure accidents were not zero. Insufficient training of participants in hygiene and and safety of patients in 37.1%. All services have seen at least one needle collector, pedal and manually opening bins.

## Conclusion

The situation in most of our developing countries deserves special attention. Indeed, the deficiencies in the management of waste and the fight against infections associated with acts of care, supply and quality unreliable drugs, inefficient staff, inadequately motivated and poorly trained and are problems in finance sector are accentuated iatrogenic problems in these countries compared to industrialized countries (WHO, 2002).

## Disclosure of interest

None declared

